# High-fiber diets attenuate emphysema development via modulation of gut microbiota and metabolism

**DOI:** 10.1038/s41598-021-86404-x

**Published:** 2021-03-26

**Authors:** Yoon Ok Jang, Ock-Hwa Kim, Su Jung Kim, Se Hee Lee, Sunmi Yun, Se Eun Lim, Hyun Ju Yoo, Yong Shin, Sei Won Lee

**Affiliations:** 1 Department of Pulmonary and Critical Care Medicine, and Clinical Research Center for Chronic Obstructive Airway Diseases, Asan Medical Center, University of Ulsan College of Medicine, 88, Olympic-ro 43-gil, Songpa-gu, Seoul, 05505 Republic of Korea; 2 Department of Convergence Medicine, Asan Medical Center, Asan Institute for Life Sciences, University of Ulsan College of Medicine, Seoul, Republic of Korea; 3 Department of Pulmonology, Allergy and Critical Care Medicine, CHA Bundang Medical Center, CHA University, Seongnam-si, Republic of Korea; 4 Metagenome Service Department, Macrogen, Inc., Seoul, Republic of Korea; 5 Department of Biotechnology, College of Life Science and Biotechnology, Yonsei University, 50 Yonsei-ro, Seodaemun-gu, Seoul, Republic of Korea

**Keywords:** Molecular biology, Molecular medicine

## Abstract

Dietary fiber functions as a prebiotic to determine the gut microbe composition. The gut microbiota influences the metabolic functions and immune responses in human health. The gut microbiota and metabolites produced by various dietary components not only modulate immunity but also impact various organs. Although recent findings have suggested that microbial dysbiosis is associated with several respiratory diseases, including asthma, cystic fibrosis, and allergy, the role of microbiota and metabolites produced by dietary nutrients with respect to pulmonary disease remains unclear. Therefore, we explored whether the gut microbiota and metabolites produced by dietary fiber components could influence a cigarette smoking (CS)-exposed emphysema model. In this study, it was demonstrated that a high-fiber diet including non-fermentable cellulose and fermentable pectin attenuated the pathological changes associated with emphysema progression and the inflammatory response in CS-exposed emphysema mice. Moreover, we observed that different types of dietary fiber could modulate the diversity of gut microbiota and differentially impacted anabolism including the generation of short-chain fatty acids, bile acids, and sphingolipids. Overall, the results of this study indicate that high-fiber diets play a beneficial role in the gut microbiota-metabolite modulation and substantially affect CS-exposed emphysema mice. Furthermore, this study suggests the therapeutic potential of gut microbiota and metabolites from a high-fiber diet in emphysema via local and systemic inflammation inhibition, which may be useful in the development of a new COPD treatment plan.

## Introduction

Chronic obstructive pulmonary disease (COPD) is a progressive respiratory disease characterized by persistent airflow obstruction and abnormal inflammatory responses^1,2^. Cigarette smoking (CS) is a major risk factor for respiratory and systemic diseases including COPD, cardiovascular disease, and cancer due to the pathogenicity of circulating toxic constituents, metabolites and their associated inflammatory response^3,4^. Moreover, CS can directly or indirectly influence the microbiome through immune homeostasis, biofilm formation, oxygen deprivation, and other potential mechanisms^5,6^.

Many studies have revealed that the gut microbial community (gut microbiota) affects metabolic function, inflammation, and physiological processes that modulate local and systemic inflammatory responses. Several scientific findings have highlighted the correlation between the gut microbiota and lung immunity in the gut-lung axis^7,8^. The constituents of the gut microbiota change in response to dietary nutrients, which are associated with immune responses and homeostasis. This interaction between gut microbiota and nutrients is also associated with respiratory diseases, mostly studies in asthma and allergy, along with gut microbiome dysbiosis^9,10^. Among nutrients working as prebiotics, dietary fibers are associated with a more diversified gut microbiota that releases metabolites with multiple functions in the host, and they are known to reduce the risk of chronic inflammatory diseases.

The metabolites produced by the gut microbiome regulate the host’s immune system and influence various organs. In accordance with these findings, the physiological influence of gut microbiota alteration and subsequent nutrient variation are governed by the differences in microbial metabolome profiles. Furthermore, metabolic byproducts derived from the bacterial fermentation of dietary fibers are important local and systemic signaling molecules that sustain immune and tissue homeostasis^11^. Among them, short-chain fatty acids (SCFAs) are metabolites that have been studied extensively, and they have immunomodulatory functions within the diverse aspects of host physiology^12^. SCFAs are produced by the fermentation of dietary fiber by specific microbes in the intestinal colon and exhibit anti-inflammatory properties^13,14^. Bile acids (BAs) are essential components of the digestive system and it reflects the amount of those escaping extraction from the portal blood^15^. Interestingly, they have also been associated with lung injury^16–19^ and lung inflammation^20–22^. Sphingolipids (SLs) can be rapidly generated or converted into each other, and they play pivotal roles in various cellular processes^23^. Moreover, SLs are increasingly recognized as critical mediators in major pulmonary diseases and are associated with COPD phenotypes^23,24^. Among them, ceramide, an intermediate product of sphingomyelin (SM) metabolism, is associated with COPD^25^ and the inflammatory response^26^.

In accordance with these findings, gut microbiota, metabolites, and nutrients are closely associated with the increased incidence of various inflammatory diseases. We previously performed a study to show that fecal microbiota transplantation and high fiber diet attenuated emphysema development and changed the gut microbiota composition^27^. Despite the growing knowledge on the immunomodulatory effects of the gut microbiota on systemic diseases, microbiota-metabolite interaction induced by dietary nutrients in the gut-lung axis in emphysema needs to be investigated further. Therefore, in this study, we investigated the effect of high-fiber diet on emphysema and the impact of gut microbiota-metabolite interaction by different types of dietary fiber compositions in a CS-exposed emphysema model. Furthermore, the relationship between the gut microbiota and metabolites was investigated based on the high-fiber diet components by analyzing the altered microbiota and metabolome profiles in a CS-exposed emphysema model.

## Results

### Dietary fiber improves alveolar destruction and inflammation during emphysema progression

**Figure 1:**
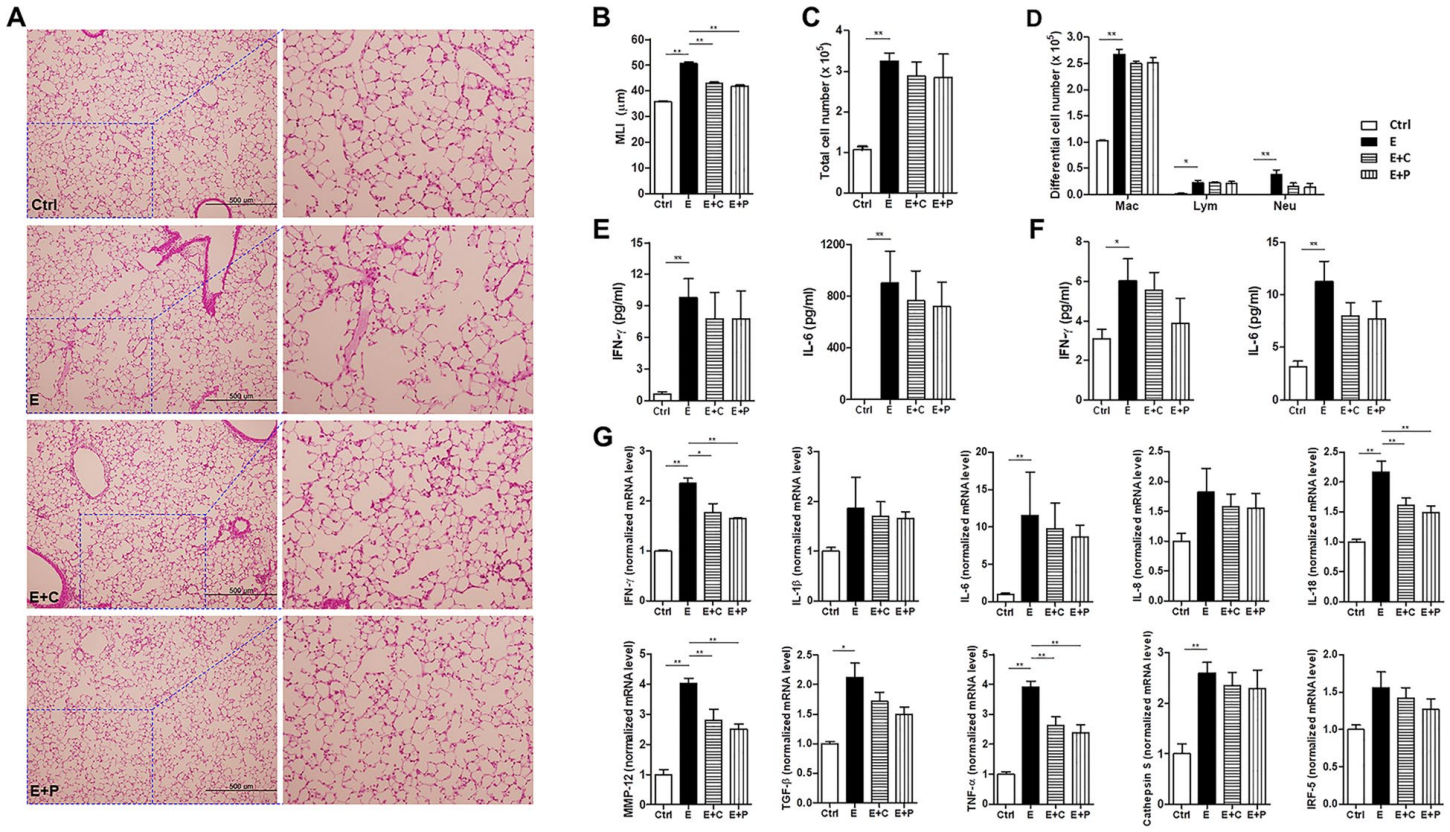
High-fiber diet attenuates inflammation and the degree of alveolar destruction. (**A**) Representative H&E-stained lung tissues from mice in the four groups (magnification: ×100 and ×400). (**B**) The mean linear intercept (MLI) of lung tissues from each group. (**C**) Total number of cells in the bronchoalveolar lavage fluid (BALF) infiltrating the airways. (**D**) Differential cell numbers of BALF in each group. (**E**,**F**) The levels of the cytokines IL-6 and IFN-γ in the BALF (**E**) and serum (**F**) were measured using ELISA. (**G**) The relative mRNA levels of IFN-γ, IL-1β, IL-6, IL-8, IL-18, IRF-5, MMP-12, TNF-α, TGF-β, and cathepsin S in lung tissues. (n = 5–6 mice per group). Values are expressed as the mean ± SE. *P < 0.05 and **P < 0.01. *Ctrl* control group, *E* emphysema group, *E* + *C* emphysema with high-cellulose diet group, *E* + *P* emphysema with high-pectin diet group.

To determine whether dietary fiber influence emphysema, the effect of high-fiber dietary modification on pulmonary inflammatory responses in CS-exposure-induced emphysema was first analyzed. Histological analysis showed that alveolar destruction and airspace enlargement were most severe in the emphysema group and less severe in the emphysema with high-fiber diet group (Fig. 1A). This observation was confirmed by the mean linear intercept (MLI) measurement, which was significantly lower in the emphysema with high-fiber diet group than in the emphysema group (Fig. 1B). The alveolar structures and forms of histological emphysema were more improved in the high-pectin diet group compared to those in the high-cellulose diet group (Fig. 1A,B). Moreover, the bronchoalveolar lavage fluid (BALF) for immune infiltration to the inflammatory response was analyzed. Inflammatory cell infiltration was higher in the BALF of all CS-exposed groups (Fig. 1C). This inflammation was characterized by increased macrophage infiltration, which was the highest in the emphysema group. The number of macrophages and neutrophils in the BALF was significantly lower in the emphysema with high-fiber diet group than in the emphysema group (Fig. 1D). The results of the analysis of the inflammatory cells from the BALF indicated a marginal decrease in the number of macrophages and neutrophils in the high-pectin diet group compared to the high-cellulose diet group. Moreover, the inflammatory cytokine levels in the BALF and serum were further evaluated. The levels of interleukin-6 (IL-6) and interferon-γ (IFN-γ) were significantly increased in the BALF and serum of the emphysema group, which is presumably associated with increased macrophage infiltration. The IL-6 and IFN-γ levels in the BALF and serum were lower in the high-pectin diet group than those in the emphysema group (Fig. 1E,F). The inflammatory cytokine levels in the BALF and serum were lower in the high-pectin diet group than those in the high-cellulose diet group. In accordance with the above results, the mRNA expression levels of representative pro-inflammatory mediators and immune modulators, including IFN-γ, IL-1β, IL-6, IL-8, IL-18, IRF-5, MMP-12, TNF-α, TGF-β, and cathepsin S, were lower in the emphysema with high-fiber diet group than those in the emphysema group. The expression of inflammatory markers was lowest in the high-pectin diet group than in all other groups (Fig. 1G). These results indicated that the high-fiber diet attenuated the pathological changes during emphysema progression and influenced an inflammatory response associated with CS-exposed emphysema. Moreover, less inflammation was observed in emphysema mice supplemented with fermentable fiber content (pectin) than in emphysema mice supplemented with non-fermentable fiber content (cellulose). Furthermore, these results indicate that a prebiotic high-fiber diet helps to prevent CS-exposure-induced emphysema progression and suggests that immune modulation by dietary fibers can attenuate the local and systemic effects of emphysema.

### Dietary fiber modulates the microbial community structure

**Figure 2:**
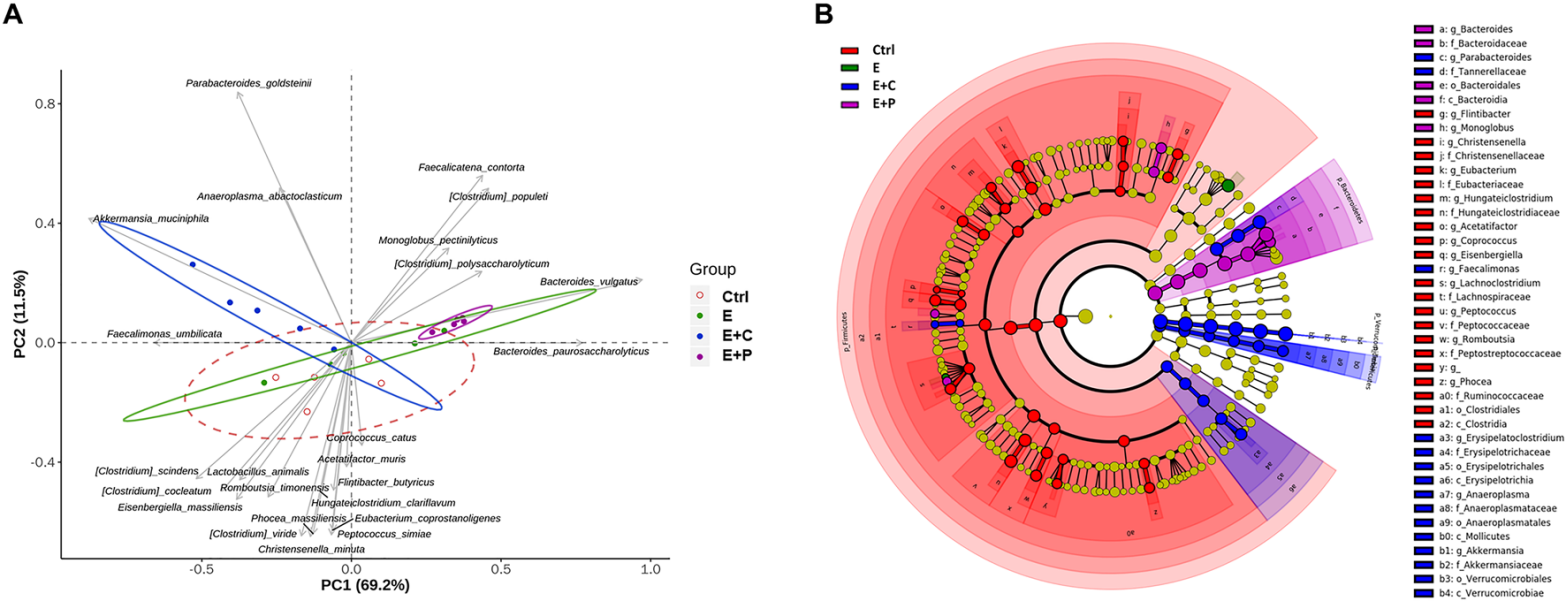
High-fiber diet alters the composition of the gut microbial community. (**A**) Principal coordinate analysis (PCoA) biplot of the fecal microbiota composition. (**B**) The cladogram of the linear discriminant analysis effect size (LEfSe) analysis for differentially abundant taxa of fecal microbiotas in the four groups; Ctrl (red), control group; E (green), emphysema group; E + C (blue), emphysema with high-cellulose diet group; E + P (violet), emphysema with high-pectin diet group. (n = 5 mice per group).

**Figure 3:**
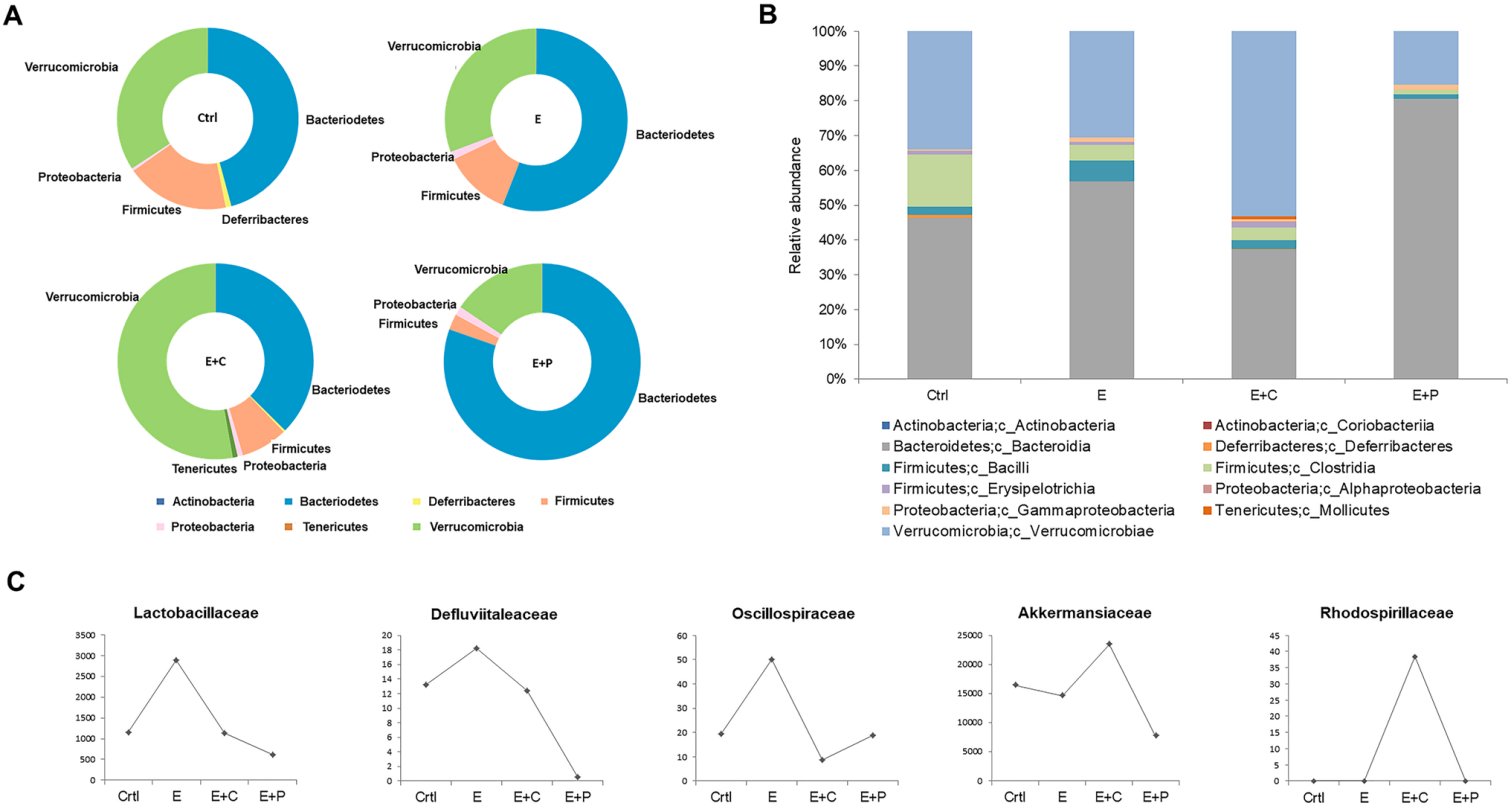
High-fiber diet changes the gut microbiota composition. Taxonomic plots showing the relative abundance across the different groups. (**A**,**B**) Phylum- (**A**) and class- (**B**) level relative abundance of the fecal microbiome of each group. (**C**) Family level bacteria-specific relative compositional changes in all groups. (n = 5 mice per group). *Ctrl* control group, *E* emphysema group, *E* + *C* emphysema with high-cellulose diet group, *E* + *P* emphysema with high-pectin diet group.

Diet has a major impact on gut microbiota composition, diversity, and richness. Different components of diet shape the gut bacterial communities. Considering that different dietary fiber components have different impacts on emphysema, different types of dietary fiber components were hypothesized to influence the microbial composition and are related to the immunity in the gut-lung axis. To confirm this hypothesis, the microbial analysis of the fecal samples was performed. A principal coordinate analysis based on the relative abundance of genera represents the distinct fecal microbial community structures. Different components of dietary fiber were significantly associated with different microbial community structures in the gut (Fig. 2A). Moreover, changes in the microbial composition of the fecal samples were analyzed using the linear discriminant analysis effect size (LEfSe) cladogram (Fig. 2B) and histogram (Fig. S1). Furthermore, the differences in the microbial composition were evaluated based on the phylum, class, and family levels (Fig. 3A-C). The Bacteroidetes phylum level was the most abundant in the emphysema with high-pectin diet group compared to the other groups (Fig. 3A). At the family level, pyrosequencing showed that the emphysema group had increased *Lactobacillaceae*, *Defluviitaleaceae*, and *Oscillospiraceae* family members compared to the control group. On the other hand, the high-fiber diet group had decreased amounts of *Lactobacillaceae*, *Defluviitaleaceae*, and *Oscillospiraceae* compared to the emphysema group and *Lactobacillaceae* and *Defluviitaleaceae* family levels were the lowest in the high-pectin diet group (Fig. 3C). The emphysema with high-cellulose diet group had the highest abundance of the Verrucomicrobia phyla (genus *Akkermansia*) and Alphaproteobacteria class (Fig. 3A). Furthermore, within the Alphaproteobacteria class, the *Rhodospirillaceae* family was observed only in the high-cellulose diet group (Fig. 3C). Based on these findings, it is observed that different types of dietary fiber components can regulate the changes in the gut microbial composition. These results provide a mechanistic insight into the protective nature of dietary-modulated microbiota during the progression of emphysema and suggest a beneficial effect on dietary-microbiota-immunity.

### Dietary fiber regulates the global metabolomic profile

**Figure 4:**
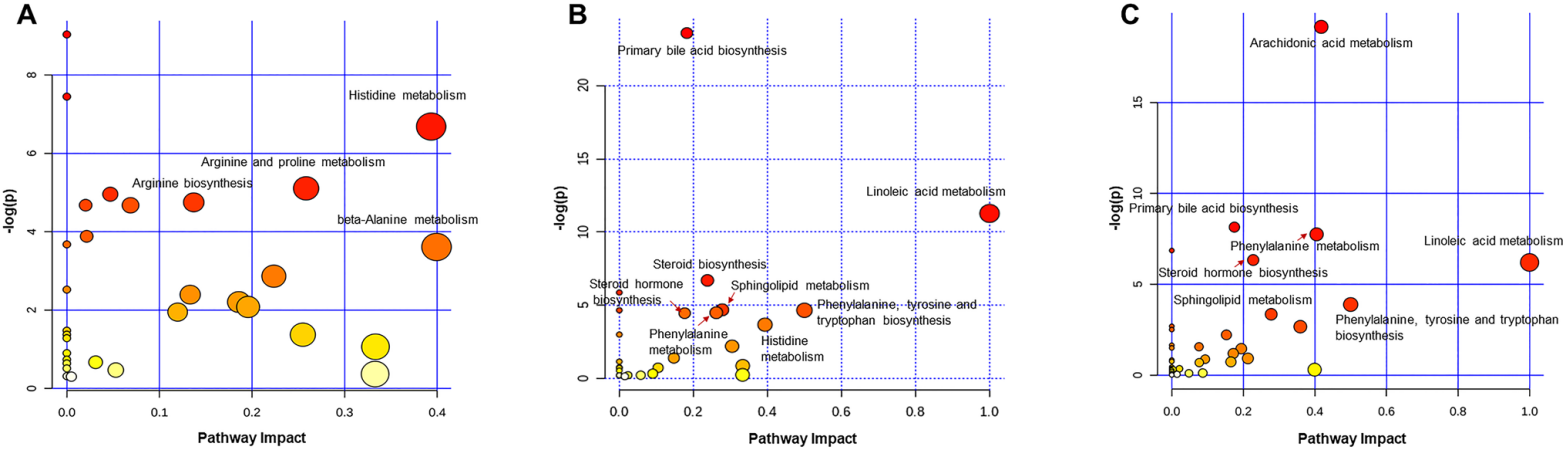
Summary of pathway analysis with MetaboAnalyst. (**A**–**C**) Metabolic pathway analysis of the feces in E (**A**), E + C (**B**), and E + P (**C**) were performed with significantly changed metabolic features (*P* < 0.05). Potential target pathways were labeled based on the impact-value threshold (0.10) and *P*-value (0.05). *Ctrl* control group, *E* emphysema group, *E* + *C* emphysema with high-cellulose diet group, *E* + *P* emphysema with high-pectin diet group.

To confirm the association between metabolites and emphysema or fiber, global metabolomic profiles were analyzed using MetaboAnalyst 4.0. The four groups in the feces, serum, and lung were classified based on the partial least squares discriminant analysis (PLS-DA). The optimal number of metabolic features for classification was selected based on the best predictive ability of the model. Five unidentified metabolites in the feces, three unidentified metabolites and L-phenylalanine in the serum, and one unidentified metabolite and AMP in the lung were used as basis for the classification, respectively (Fig. S2). A pathway analysis was performed using a KEGG library with significantly changed metabolic features. Based on the *P* value < 0.05 in the pathway analysis and impact value threshold (0.10), various pathways induced by CS-exposed emphysema and fiber diet were identified. The comparison between the two groups was further analyzed in three ways: (1) control vs. emphysema, (2) emphysema vs. emphysema with high-cellulose diet, and (3) emphysema vs. emphysema with high-pectin diet for feces, affected by microbiota ecology primarily. β-alanine metabolism and histidine metabolism had the highest impact (0.40) on grouping control and emphysema. Several common pathways were significantly affected by high-cellulose and -pectin diet. Linoleic acid, primary BA synthesis, phenylalanine, tyrosine and tryptophan biosynthesis, steroid hormone biosynthesis, and SL metabolisms exhibit a significant effect. Among them, linoleic acid metabolism showed the highest impact value (1.0) in both comparisons (Fig. 4), and primary BA biosynthesis had the most significant impact with a *P* value of 2.28 × 10^–6^. Linoleic acid metabolism and d-glutamate metabolism showed the highest impact values (1.0) in the lungs based on the pathway topology analysis (Fig. S3). Caffeine metabolism and linoleic acid metabolism had the highest impact in the serum. Arachidonic acid metabolism was the pathway with significant impact in both feces (Fig. 4) and serum (Fig. S4). The metabolic patterns provide an overview of the metabolic differences in the four groups. The top metabolites representing the differences in the feces, lung, and serum are visually illustrated by heatmaps (Fig. S5). Furthermore, the targeted metabolites were different in the four groups.

### Dietary fiber regulates targeted metabolomic profiles

**Figure 5:**
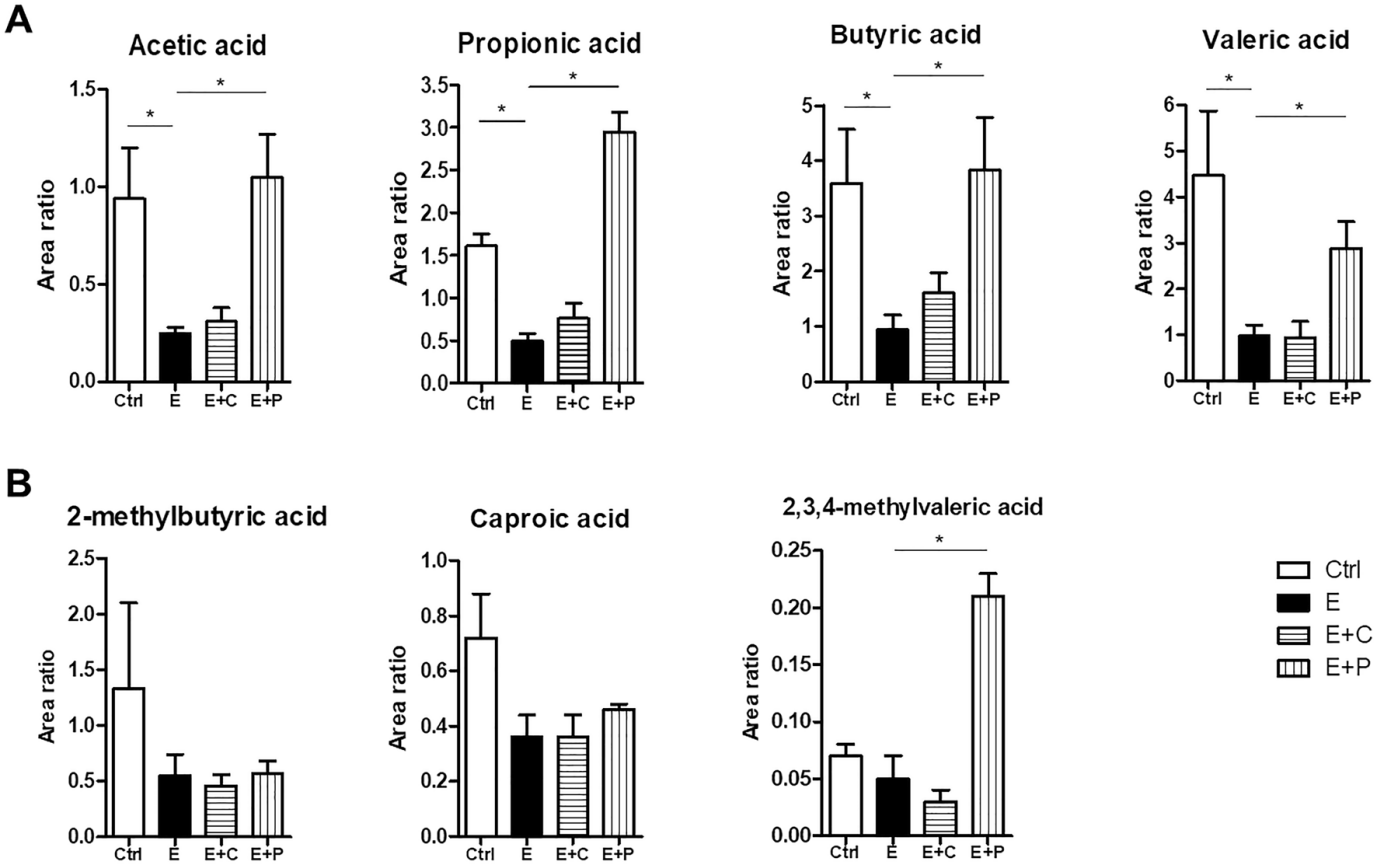
Dietary fiber regulates the short chain fatty acid (SCFA) metabolism. (**A**,**B**) The concentrations of the SCFA metabolites (acetic, propionic, butyric, valeric, 2-methylbutyric, caproic, and 2,3,4-methylvaleric acids) were measured within the content of the fecal metabolite profiling. (n = 4 mice per group; n = 5 mice in E + P). Values are expressed as the mean ± SE. *P < 0.05 and **P < 0.01. *Ctrl* control group, *E* emphysema group, *E* + *C* emphysema with high-cellulose diet group, *E* + *P* emphysema with high-pectin diet group.

**Figure 6:**
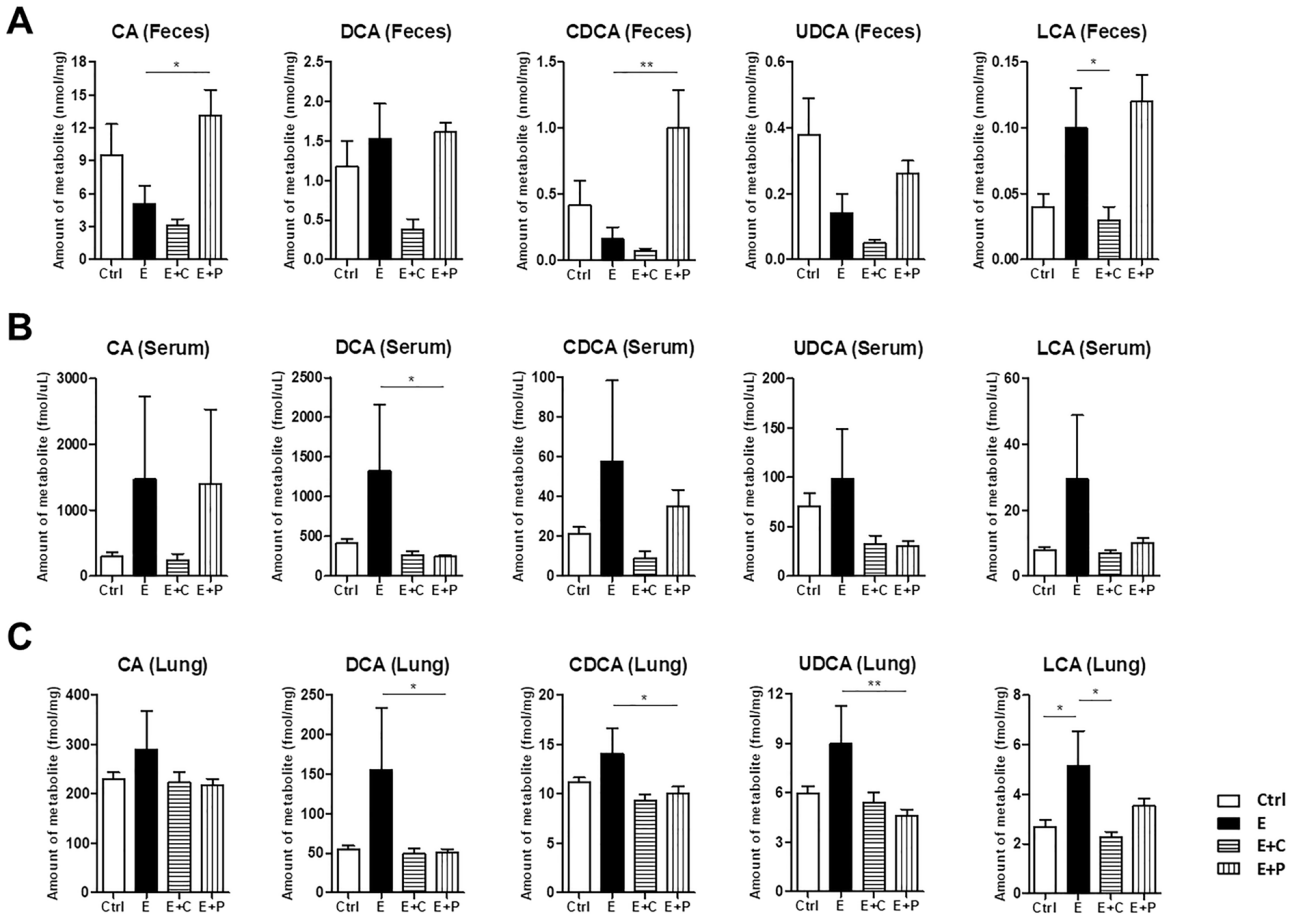
Dietary fiber alters the bile acid (BA) metabolism. (**A**–**C**) The levels of the BA metabolites (CA, DCA, CDCA, UDCA, and LCA) in the feces (**A**), serum (**B**), and lung (**C**) were measured using metabolite profiling. (n = 6 mice per group). Values are expressed as the mean ± SE. *P < 0.05 and **P < 0.01. *Ctrl* control group, *E* emphysema group, *E* + *C* emphysema with high-cellulose diet group, *E* + *P*, emphysema with high-pectin diet group, *CA* cholic acid, *DCA* deoxycholic acid, *CDCA* chenodexycholic acid, *UDCA* ursodeoxycholic acid, *LCA* lithocholic acid.

Further analysis with targeted metabolomic profiles was performed for SCFAs, BAs, and SLs, and detailed results were described in the supplementary results. Overall, the acetate, propionate, and butyrate concentrations were the lowest in the emphysema group compared to the other groups. The local concentrations of SCFAs (acetate, propionate, and butyrate) were notably higher in the emphysema with high-fiber (cellulose and pectin) diet group than in the emphysema group. The SCFA (acetate, propionate, and butyrate) concentrations were increased in the high-pectin diet group compared to the high-cellulose diet group (Fig. 5). The results are similar to those previously reported^28^. The primary BA concentrations, such as cholic acid (CA) and chenodeoxycholic acid (CDCA), in the feces were higher in the control group than in the emphysema group (Fig. 6A). The secondary BA concentrations, such as deoxycholic acid (DCA) and lithocholic acid (LCA), in the feces, serums, and lung tissues were higher in the emphysema group than in the control group (Fig. 6A-C). Interestingly, the CA, CDCA, and ursodeoxycholic acid (UDCA) concentrations in the feces were higher in the high-pectin diet group than in the emphysema group. Meanwhile, the CDCA, DCA, LCA, CA, and UDCA concentrations in the lung tissues and serums were higher in the emphysema group than in the other groups (Fig. 6). These results are similar to those previously reported in respiratory disease^29^.

## Discussion

The gut microbiota, via various dietary nutrients, is involved in the production of metabolites that play a major role in human immunity. Metabolites like SCFAs significantly influence systemic immunity and the circulatory systems^30^. Therefore, it is reasonable that gut microbiota dysbiosis via malnutrition or unhealthy diet is associated with dysregulated inflammation affecting autoimmune or respiratory diseases^31^. However, the interactions between dietary fiber components and the regulatory mechanisms of respiratory diseases have not been investigated.

This study explored whether dietary fibers acting as prebiotics directly interact with gut microbes and play a role in the production of key metabolites, including SCFAs, BAs, and SL. This study also discussed how dietary fibers impact gut microbial ecology, host physiology, and immunity. The therapeutic potential of modulating gut microbiota and metabolites by a high-fiber diet was investigated in a CS-exposed emphysema model. CS is a major risk factor for COPD, which is impacted by the significant pathogenicity of circulating toxic constituents or metabolites of absorbed CS^4,32^. A CS-induced emphysema model was used based on general pathologies and mechanisms of human emphysema. In this model, a high-cellulose and high-pectin diet significantly suppressed the inflammation and attenuated the pathological changes in emphysema mice. The number of immune cells, macrophages, and neutrophils was lower in the high-fiber (cellulose and pectin) diet group than in the emphysema group. These results confirm that inflammatory cytokine levels and mRNA levels were decreased in the high-fiber (cellulose and pectin) diet group and that dietary fiber strongly inhibits inflammation. The effect of high fiber diet on lung function and COPD is also proven in epidemiologic studies^33,34^, although the underlying mechanism is not clear. Extensive studies have found that the gut microbiota is crucial in maintaining host homeostasis via immune system interactions^35,36^. Low gut microbial diversity is associated with increased risk of lung disease^37^. The interactions between dietary fiber components and gut microbial communities were investigated in an emphysema model. The present study showed that the emphysema group had increased *Lactobacillaceae*, *Defluviitaleaceae*, and *Oscillospiraceae* family members compared to the control group. The high-cellulose and high-pectin diet groups had lower *Lactobacillaceae*, *Defluviitaleaceae*, and *Oscillospiraceae* family members compared to the emphysema group. The increase of *Lactobacillaceae* in COPD was also identified in human studies and it was also associated with local inflammation^38,39^. The role of the *Defluviitaleaceae* and *Oscillospiraceae* family members (Clostridia class) in the respiratory diseases has not yet been investigated, our study suggests the necessity to further confirm their roles. Interestingly, the *Rhodospirillaceae* family (Alphaproteobacteria class) was observed only in the high-cellulose diet group. Alphaproteobacteria is a diverse class of organisms within the Proteobacteria phylum with many biological roles and cellulose usually increases their numbers^40,41^. Bacteroidetes are major producers of SCFAs, and the proportion of Bacteroidetes is significantly decreased in cases of COPD^42^. Accordingly, the emphysema with high-pectin diet group had the highest abundance of the Bacteroidetes phyla compared to the other groups in this study. The emphysema with high-cellulose diet group had the highest abundance of the Verrucomicrobia phyla (genus *Akkermansia*) and the Alphaproteobacteria class (phylum Proteobacteria), which has many important biological roles. These data indicate that both pectin and cellulose supplementation modulates the microbial community structures and effectively alter the composition of the gut microbiota. The protective effects of high-fiber diet against emphysema progression may be influenced by the direct interactions of microbiota, dietary fiber and immune system.

Various metabolites have been extensively investigated in terms of their beneficial immunomodulatory roles^43,44^. Specifically, SCFAs reduce colitis by inducing colonic regulatory T cells^45^ and driving colonocytes towards mitochondrial beta oxidation of fatty acids^46^. SCFAs also prevent asthma pathology^28^. Malnutrition adversely affects the gut microbiota composition and decreases the production of physiologically active byproducts that are key driving factors in the prevalence of inflammatory disease^44^. Previous studies have reported that dietary fiber-induced microbial SCFAs not only suppress the ILC2-dependent airway inflammation^47^ but also modulate immunity^28^. Similarly, in the present study, the feces from the emphysema with high-fiber (cellulose and pectin) diet group were significantly enriched with acetate, propionate, and butyrate and had large alterations in the gut microbiota. Moreover, the SCFAs concentrations were increased in the high-pectin diet group than in the high-cellulose diet group. Moreover, the valeric acid and 2,3,4-methylvaleric acid concentrations were higher in emphysema mice with high-pectin diet than in emphysema mice. We confirmed that high-pectin diet was associated with an abundance of SCFA with anti-inflammatory properties in an emphysema model. These results show that different types of dietary fiber ingredients (fermentable or non-fermentable) induce different changes in metabolite levels and regulate the levels of different SCFA metabolites. In addition, the protective effects of dietary fiber induced SCFAs through the interactions between the microbiota and immune system may inhibit the progression of emphysema.

Among the various endogenous metabolites produced by host-gut microbiota metabolism, BAs are gaining increased attention^48^. They serve as nutrient signaling hormones, play a role in inflammation, and are associated with COPD and lung disease inflammation^16–20^. We also explored the regulation of BA metabolites by different dietary fiber components in an emphysema model. In this study, we showed that the DCA and LCA concentrations in the feces, serums, and lung were higher in emphysema mice than in control mice. Moreover, the DCA, LCA, and CDCA concentrations in serums and lung were lower in the high-pectin diet and high-cellulose diet groups than in the emphysema group. The secondary BA concentrations (DCA, UDCA, and LCA) in the feces were higher in the high-pectin diet group compared to other groups. A previous review article suggested that dietary fibers bind conjugated primary BAs and may serve as a platform for gut bacteria, thus leading to deconjugation which is a perquisite for biotransformation to secondary BAs^49^. Therefore, this indicates that different types of dietary fiber components can affect various BA metabolites. Moreover, this study suggests that BAs, via dietary-metabolic regulation, are associated with local and systemic inflammatory responses in an emphysema model.

SLs are constituents of cellular membranes and participate in cellular responses to stress, thereby ensuring cell survival. Therefore, the proper functioning of the SL metabolic pathway is essential for cellular homeostasis^50^. Moreover, the role of SLs as mediators of inflammation may have significant implications in a range of pulmonary diseases in which inflammation is a central process of the pathogenesis^51^. The extensive alterations in SL metabolism induced by CS exposure are controlled by a complex regulatory program, which, concomitantly, may also mediate other adaptive and destructive tissue responses. Moreover, changes in SL metabolism are intrinsic to the broad response of the tissue to CS exposure and dietary fiber intake, including oxidative stress and xenobiotic stress responses, immune response, and other metabolic alterations. As smoking-related inflammation and a high-fiber diet impact SL metabolism, the association between SLs and inflammation in COPD can be determined. Further metabolomics studies are needed to investigate the potential diagnostic and therapeutic candidates in emphysema.

The fatty acids including alpha-linolenic acid and linoleic acid are essential for humans^52^. Omega-3 fatty acids, such as plant-derived alpha-linolenic acid and its forms primarily found in fish, give rise to anti-inflammatory and pro-resolving mediators^53^. Previous studies have reported that the higher dietary intake of omega-3 fatty acids leads to the decrease in cardiovascular risk/atherosclerosis^54^, autoimmune disease activity^55^, morbidity in asthma^56^, COPD^57^, and other chronic diseases^58^. Global metabolomics profile analysis revealed that the linoleic acid metabolism pathway had the highest impact on the emphysema with high-cellulose and high-pectin diet groups. Therefore, these results suggest that anti-inflammatory mechanisms including the linoleic acid pathway prevent the COPD development.

These results suggested that the protection exhibited by high-cellulose or high-pectin diet against emphysema may depend on the microbiota-metabolism-immunity interactions. However, the complex relationship among these biological systems and prebiotics in emphysema needs further investigation. Additionally, the bioavailability of nutrients and bioactive compounds in fruits and vegetables is an extremely important area of nutrition and medical research. Water-soluble dietary fibers can impair the bioavailability of beta-carotene in the gastrointestinal tract^59–62^. Thus, further research is required to understand the dietary-fiber-associated bioavailability and functions of carotenoids.

The important regulatory roles of naturally occurring deuterium in fundamental biological processes and the antitumor effect of deuterium depletion have been demonstrated in multiple studies^63–67^. Deuterium depletion occurs through returning low deuterium ketogenic propionic acid to the circulation for terminal oxidation into CO_2_ and deuterium-depleted metabolic water in the mitochondria/peroxisomes of host cells^68^. Hence, the correlation among emphysema, deuterium-depleted metabolites, and metabolizing organisms in the gut microbiome-SCFAs digesting dietary fiber needs to be investigated further. New insights into the role of deuterium depletion in emphysema may facilitate the development of novel emphysema treatments.

The findings of this study indicate that compositions of the gut microbiota and metabolites can be modulated using a high-fiber diet, consequently attenuating emphysema development via inhibition of local and systemic inflammation, which may provide future therapeutic applications for preventing or delaying emphysema progression.

## Materials and methods

### Emphysema mouse model

Female C57BL/6 mice (8 weeks old) were purchased from Orient Bio Inc. (Seongnam, Republic of Korea) and maintained at room temperature (RT, 25 °C) in a 12/12-h light/dark cycle. The animal model of emphysema was exposed with CS according to a previously reported protocol^27^. This study complied with guidelines of the 8th edition of the Guide for the Care and Use of Laboratory Animals. All animal experiments were approved by the Institutional Animal Care and Use Committee of Asan Medical Center, Seoul, Republic of Korea (2019-14-069), and were performed according to the ARRIVE (Animal Research: Reporting In vivo Experiments) guidelines^69^.

### Diet modifications

The animals were randomly divided into four groups (n = 6 mice/group): Group I (control group, Ctrl), Group II (emphysema group, E, CS exposure only), Group III (E + C, CS exposure with a high-cellulose diet), and Group IV (E + P, CS exposure with a high-pectin diet). The mice were fed ad libitum with the standard AIN-76A diet supplied by Daehan Biolink Co., LTD (Chungbuk, Republic of Korea). The two types of dietary modifications based on the standard AIN 76A diet were as follows: the high-cellulose diet (20% cellulose), which was modified with cellulose from vegetable supplement, and the high-pectin diet (20% pectin), which was modified with pectin from citrus supplement. Groups I and II were given the standard AIN-76A diet for 4 weeks. Group III and IV were given the AIN-76A diet for 1 week and were administered with a modified diet for the last 3 weeks of the experiment period.

### Preparation of samples

After 4 weeks, the animals were anesthetized through isoflurane inhalation, and the blood samples were collected by heart puncture. The trachea was catheterized and perfused with 1.5 mL of PBS. The cellular and liquid fractions of the BALF were separated by centrifugation at 2200 rpm for 5 min at 4 °C. The cell pellet was suspended in PBS, seeded onto a slide, and stained with Diff-Quick (Sysmex, Kobe, Japan). Inflammatory cells were observed under a light microscope (Leica, USA). All specimens were collected, fixed, immediately frozen, and stored at − 80 °C until analysis.

### Histomorphological assessment

The left lung was inflated with 0.5% low-melting-point agarose at a pressure of 15 cm H_2_O. The left lobe was embedded in paraffin, cut into 5-μm-thick sections, and stained with hematoxylin and eosin. Emphysematous changes were observed under a light microscope (Leica, USA) and were evaluated by measuring the MLI. MLI is a measurement of the mean interalveolar septal wall distance determined by the number of interruptions in 1 mm lines of the alveolar wall. Four lines were drawn in each field, and at least five random fields were examined per mouse.

### Cytokine level quantification

The levels of the cytokines IL-6 and IFN-γ in the BALF and serum were measured using a commercially available ELISA kit (R&D Systems, Minneapolis, MN, USA) based on the manufacturer’s instructions.

### Quantitative real-time PCR analysis

Total RNA was extracted from lung tissues using TRIzol reagent (Thermo Fisher Scientific, Waltham, MA, USA) in accordance with the manufacturer’s protocol as previously described^27^. Transcription levels were measured using real-time PCR with sequence-specific primers for IFN-γ, IL-1β, IL-6, IL-8, IL-18, MMP-12, TNF-α, TGF-β, cathepsin S, and IRF-5 (Table S1).

### DNA extraction and microbiota bacterial composition analysis

DNA was extracted from fecal samples using a DNA isolation kit (MO BIO, Carlsbad, CA, USA) in accordance with the manufacturer’s instructions. DNA was amplified with 16S_V3_F and 16S_V4_R primers specific to the V3-V4 hypervariable regions of the 16S rDNA gene (Supplementary Table S1). The libraries were prepared using PCR products in accordance with the MiSeq System Guide (Illumina, USA) and quantified using the QIAxpert System (QIAGEN, Germany). Each amplicon was quantified, and an equimolar ratio was pooled and sequenced on a MiSeq (Illumina) in accordance with the manufacturer’s instructions. Microbiota bacterial composition analysis was performed, following the procedure previously described^27^.

### Quantitative metabolome measurement

Standard metabolites and internal standards were purchased from Sigma-Aldrich (St. Louis, MO, USA), and all solvents were purchased from J. T. Baker (Center Valley, PA, USA). The SCFA metabolites in feces and BA metabolites were measured using a previously described procedure^70,71^. Mouse serum (20 µL) was mixed well with 500 μL of cold acetonitrile and 50 μL of internal standard solution (50 μL of 1 μM cholic acid-d_5_ solution). For the SL metabolites, the Folch method was used to extract the lipids from 20 to 30 mg of mouse lung after adding 50 μL of internal standard solution (50 nM of C17 ceramide solution)^72^. For the 30 μL of mouse serum, the lipids was extracted using the Bligh and Dyer method after adding the internal standard solution^73^. The organic layer containing sphingolipids was dried using a vacuum centrifuge and stored at − 20 °C until liquid chromatography-tandem mass spectrometry (LC–MS/MS) analysis. The dried matter from the feces, serum, and lung was stored at − 20 °C and reconstituted with methanol (MeOH) prior to LC–MS/MS analysis for all the analyses performed. Furthermore, the samples were analyzed using a LC–MS/MS system equipped with a 1290 HPLC (Agilent, Waldbronn, Germany) and QTRAP5500 mass spectrometry (AB Sciex, Toronto, Canada). Additional methods are described in the data supplement.

### Global metabolome profiling

The metabolites were extracted using conventional liquid–liquid extraction procedures^72,73^. Briefly, 3–4 volumes of chloroform/methanol (2/1, v/v) were added to freeze-dried feces, lung, and serum and then centrifuged for 15 min. Nonpolar metabolites containing lipids were collected from the lower organic phase, and polar metabolites were collected in the upper aqueous phase. The LC–MS was equipped with Ultimate3000 (Dionex) and Orbitrap XL (Thermo Fisher). A reverse phase column (Pursuit 5; 150 × 2.0 mm) and HILIC (HILIC Plus; 100 × 2.1 mm) were used for nonpolar and polar metabolites, respectively. LC–MS analysis was conducted for each sample solution in positive and negative ion modes. Data normalization was performed using log transformation and auto-scaling. Metabolite features, including neutral mass and retention time values, were determined using the Compound Discoverer 2.1. Moreover, metabolite features showing statistically significant changes (*P* < 0.05) were chosen and identified using the KEGG database (http://www.kegg.jp/kegg/kegg1.html) with a mass accuracy of 10 ppm. Statistical analyses including the PLS-DA were conducted using MetaboAnalyst 4.0.

### Statistical analysis

Data were analyzed using the Kruskal–Wallis *H* test, Mann–Whitney *U* test, and one-way ANOVA followed by Tukey’s test with the IBM SPSS version 25 (https://www.ibm.com/analytics/spss-statistics-software). All values were expressed as the mean ± standard error (SE). Statistical significance was set at *P* < 0.05.

## Supplementary Information

Supplementary Information
