# Supplementary material for: High-fiber diets attenuate emphysema development via modulation of gut microbiota and metabolism
Source: Sci Rep. 2021 Mar 26;11:7008. doi: 10.1038/s41598-021-86404-x (PMC7997879; doi:10.1038/s41598-021-86404-x)
Supplement: Supplementary file 1 — Supplementary Information [file 41598_2021_86404_MOESM1_ESM.docx]

**High-Fiber Diets Attenuate Emphysema Development via Modulation of Gut Microbiota and Metabolism**

Yoon Ok Jang^1,2^, Ock-Hwa Kim^1^, Su Jung Kim^2^, Se Hee Lee^1,3^, Sunmi Yun^4^, Se Eun Lim^4^, Hyun Ju Yoo^2^, Yong Shin^5^ and Sei Won Lee^1‡^

^1^Department of Pulmonary and Critical Care Medicine, Asan Medical Center, University of Ulsan College of Medicine, Seoul, Republic of Korea

^2^Department of Convergence Medicine, Asan Institute for Life Sciences, Asan Medical Center, University of Ulsan College of Medicine, Seoul, Republic of Korea

^3^Department of Pulmonology, Allergy and Critical Care Medicine, CHA Bundang Medical Center, CHA University, Seongnam-si, Republic of Korea

^4^Metagenome Service Department, Macrogen, Inc. Seoul, Republic of Korea

^5^Department of Biotechnology, College of Life Science and Biotechnology, Yonsei University, 50 Yonsei-ro, Seodaemun-gu, Seoul, Republic of Korea

**Supplement materials and methods**

**Liquid chromatography-tandem mass spectrometry (LC-MS/MS).**

SCFA metabolites in feces were measured using a previously described procedure^1^. For BA metabolites, a reverse-phase column (Pursuit 5 C18, 150 × 2.1 mm, Agilent Technologies) was used with mobile phase A (7.5 mM ammonium acetate, pH 4 using 10 M acetic acid) and mobile phase B (5% acetonitrile in MeOH). The LC was performed at 200 µl/min and 24°C. The LC gradient is as follows: 40% of A for 0 min, 40% to 20% of A for 20 min, 20% to 10% of A for 5 min, 10% of A for 5 min, 10% to 40% of A for 5.1 min, and 40% of A for 4.9 min. For SL metabolites, a reverse-phase column (Pursuit 5 C18, 150 × 2.1 mm) was used with mobile phase A (5 mM ammonium formate/MeOH/tetrahydrofuran (500/200/300, v/v/v) and mobile phase B (5 mM ammonium formate/MeOH/tetrahydrofuran (100/200/700, v/v/v). The LC was performed at 200 µl/min and 35°C. The LC gradient is as follows: 50% of A for 0 min, 50% of A for 5 min, 50% to 30% of A for 3 min, 30% of A for 7 min, 30% to 10% of A for 7 min, 10% of A for 3 min, 10% to 50% of A for 0.1 min, and 50% of A for 4.9 min. Multiple reaction monitoring (MRM) was performed in the negative ion mode for BAs and positive ion mode for SLs. The extracted ion chromatogram corresponding to the specific transition of each BA or SL was used for quantification. The calibration range for each analyte was 0.1–10000 nM (r^2^ ≥ 0.99). The LC-MS/MS data were analyzed using Analyst 1.5.2 software (AB Sciex).

**Supplement results**

**Dietary fiber regulates SCFA metabolism.**

The gut microbiome is a microbial ecosystem having physiological effects on major immune functions^2^. Metabolites produced by the microbiome simultaneously modulate immunity and influence various organs. Moreover, previous studies have reported that dietary, host-derived, and microbiota modulated metabolites may affect numerous aspects of the host-immune response. These findings with the above results motivated us to determine whether the beneficial effects of a high-fiber diet can cause a shift in microbial composition thereby inducing changes in gut-microbiota-derived metabolites. To verify this hypothesis, we investigated whether different dietary fiber components induced metabolite production in the emphysema model. Fecal samples from each group were retrieved for metabolite analysis. SCFA concentrations were significantly different among emphysema mice and emphysema mice with high-fiber diet (Fig. 5). The main SCFAs are acetate (C2), propionate (C3), and butyrate (C4), and they constitute 95% of SCFAs. Significantly, concentrations of acetate, propionate, and butyrate were the lowest in the emphysema group compared with other groups. The local concentrations of SCFAs (C2, C3, and C4) were notably higher in the emphysema mice with high-fiber (cellulose and pectin) diet than in the emphysema mice. Also, the SCFA (C2, C3, and C4) concentrations were higher in the high-pectin diet group than in the high-cellulose diet group. The results are similar to those previously reported^3^. The concentrations of SCFAs such as valeric acid and 2,3,4-methylvaleric acid were higher in the emphysema mice with high-fiber diet than in the emphysema mice. These results showed that different dietary fiber ingredients (fermentable or non-fermentable) induced different SCFA metabolites. We also observed that high-pectin diet was associated with an increased SCFA amount in the emphysema model. Collectively, the findings indicate that different dietary fiber components are critical in generating SCFA metabolites.

**Dietary fiber alters bile acid metabolism.**

The BAs are essential in facilitating the absorption and digestion of dietary lipids, and are recognized as enteroendocrine hormones, which play critical roles in regulating several aspects of physiology within and outside the intestinal tract. Moreover, BAs greatly influence respiratory infection and inflammation and might serve as nutrient-signaling hormones. Thus, dietary ingredients correlated with SCFA abundance. We further explored BA metabolites in feces, serums, and lung tissue contents.

The concentrations of primary BAs such as cholic acid (CA) and chenodeoxycholic acid (CDCA) in feces were higher in the control mice than in emphysema mice (Fig. 6A). Notably, higher deoxycholic acid (DCA) and lithocholic acid (LCA) values are associated with increased risk of cancer^4^. Moreover, chronic aspiration of CDCA, DCA, and LCA may be a critical profibrotic factor in the pathogenesis of pulmonary fibrosis^5^. Taken together, the concentrations of secondary BAs such as DCA and LCA in feces, serums, and lung tissues were higher in the emphysema mice than in control mice (Fig. 6A-C). Interestingly, the concentrations of CA, CDCA, and ursodeoxycholic acid (UDCA) in feces were higher in the high-pectin diet group compared with the emphysema group. The concentrations of DCA and LCA in feces were the lowest in the high-cellulose diet group compared with the other groups (Fig. 6A). Additionally, the concentrations of CDCA, DCA, LCA, CA, and UDCA in lung tissue and serum were higher in the emphysema group than in other groups (Fig. 6B,C). Moreover, the concentrations of CA, CDCA, UDCA, DCA, and LCA in lung tissue and serum were lower in the high-pectin diet and high-cellulose diet groups than in the emphysema group (Fig. 6B,C). Furthermore, DCA concentrations in lung tissue and serum and CDCA concentrations in lung tissue were lower in the high-pectin diet and high-cellulose diet groups than in the control group (Fig. 6B,C). LCA and CDCA concentrations in lung tissue and serum were lower in the high-cellulose diet group than in other groups (Fig. 6B,C). These results showed that the concentrations of DCA and LCA, which are markers for an increased risk of cancer, were lower in the high-pectin diet and high-cellulose diet groups than in the emphysema group. Observably, different dietary fiber components cause different changes in BA metabolites. The results are similar to those previously reported in respiratory disease^6^.

**Dietary fiber changes sphingolipid metabolism.**

SLs are constituents of cellular membranes and participate in cellular response to stress, thereby ensuring cell survival. Therefore, the proper functioning of the SL metabolic pathway is essential for cellular homeostasis^7^. Moreover, the role of SLs as inflammatory mediators may have significant implications in various pulmonary diseases where inflammation is a central element of pathogenesis^8^. Furthermore, ceramide, an intermediate product of SM metabolism, abnormally accumulates in lung tissue and might damage endothelial defense, induces alveolar epithelial cell apoptosis, promotes inflammatory response, and cause macrophage dysfunction^9^.

SL metabolism is associated with pulmonary diseases. Thus, we investigated the relationship between SL metabolites and CS-induced emphysema model. Ceramide levels (Cer14:0, Cer16:0, Cer18:0, and Cer24:1) in lung were increased in the emphysema mice compared with control mice. Moreover, the serum levels SMs (SM16:0, SM18:0, and SM24:1), serum sphinganine (SA), serum sphingosine (SO), and lung SA were increased in the emphysema mice compared with control mice (Fig. S6). To investigate how SL metabolites changed in response to the consumption of different dietary fiber components, we further evaluated the SL metabolite levels in emphysema mice with high-fiber diet.

Regarding the change in SL metabolites following the high-cellulose diet group, the ceramide levels (Cer14:0, Cer18:0, Cer20:0, Cer24:0, and Cer24:1) in the lung, lung SMs (SM18:0, SM24:0, and SM24:1), and serum (Cer14:0, Cer16:0, Cer18:0, Cer18:1, Cer20:0, Cer24:0, Cer24:1, SM16:0, SM18:0, SM18:1, SM24:0, and SM24:1) were lower in the high-cellulose diet group than in the emphysema group. Moreover, comparing changes in SL metabolites in the high-pectin diet group, the ceramide levels (Cer18:0, Cer18:1, Cer20:0, and Cer24:0) in the lung, lung SMs (SM16:0, SM18:0, SM18:1, and SM24:0), and serum (Cer14:0, Cer20:0, Cer24:0, SM16:0, SM18:1, and SM24:0) were lower in the high-pectin diet group than in the emphysema group. Furthermore, the ceramide levels (Cer18:0, Cer20:0, and Cer24:0) in the lung, lung SMs (SM18:0 and SM24:0), lung SA, serum ceramides (Cer14:0, Cer20:0, and Cer24:0), serum SMs (SM 16:0, SM18:0, and SM24:0), serum SA, and serum SO were lower in the high-fiber (cellulose and pectin) diet groups than in the emphysema group (Fig. S6). These results are similar to those previously reported^9-12^. These results showed that SL metabolites, including ceramide, SM, SA, and SO, were associated with increased CS-exposure-induced emphysema development. Additionally, we showed that different dietary fiber ingredients (pectin and cellulose) in emphysema contributed to different SL metabolites. Moreover, SL metabolite levels were lower in emphysema mice with high-fiber and high-cellulose diet than in emphysema mice. These results suggest that high-fiber diet contributes to the SL metabolite pathway regulating the CS-exposed emphysema. However, in the CS-induced emphysema, it is difficult to identify the alteration in SL metabolites caused by the high-fiber diet. Thus, further research is required to clarify the roles of anti-inflammatory mechanisms, including arachidonic acid metabolism, in emphysema.

**References**

1 Song, H. E., Lee, H. Y., Kim, S. J., Back, S. H. & Yoo, H. J. A Facile Profiling Method of Short Chain Fatty Acids Using Liquid Chromatography-Mass Spectrometry. *Metabolites* **9**, doi:10.3390/metabo9090173 (2019).

2 Clemente, J. C., Ursell, L. K., Parfrey, L. W. & Knight, R. The impact of the gut microbiota on human health: an integrative view. *Cell* **148**, 1258-1270, doi:10.1016/j.cell.2012.01.035 (2012).

3 Trompette, A. *et al.* Gut microbiota metabolism of dietary fiber influences allergic airway disease and hematopoiesis. *Nat Med* **20**, 159-166, doi:10.1038/nm.3444 (2014).

4 Phelan, J. P., Reen, F. J., Caparros-Martin, J. A., O'Connor, R. & O'Gara, F. Rethinking the bile acid/gut microbiome axis in cancer. *Oncotarget* **8**, 115736-115747, doi:10.18632/oncotarget.22803 (2017).

5 Chen, B. *et al.* Chronic microaspiration of bile acids induces lung fibrosis through multiple mechanisms in rats. *Clin Sci (Lond)* **131**, 951-963, doi:10.1042/CS20160926 (2017).

6 Aldhahrani, A., Verdon, B., Ward, C. & Pearson, J. Effects of bile acids on human airway epithelial cells: implications for aerodigestive diseases. *ERJ Open Res* **3**, doi:10.1183/23120541.00107-2016 (2017).

7 Petrache, I. & Berdyshev, E. V. Ceramide Signaling and Metabolism in Pathophysiological States of the Lung. *Annu Rev Physiol* **78**, 463-480, doi:10.1146/annurev-physiol-021115-105221 (2016).

8 Chakinala, R. C., Khatri, A., Gupta, K., Koike, K. & Epelbaum, O. Sphingolipids in COPD. *Eur Respir Rev* **28**, doi:10.1183/16000617.0047-2019 (2019).

9 Bodas, M., Pehote, G., Silverberg, D., Gulbins, E. & Vij, N. Autophagy augmentation alleviates cigarette smoke-induced CFTR-dysfunction, ceramide-accumulation and COPD-emphysema pathogenesis. *Free Radic Biol Med* **131**, 81-97, doi:10.1016/j.freeradbiomed.2018.11.023 (2019).

10 Yang, Y. & Uhlig, S. The role of sphingolipids in respiratory disease. *Ther Adv Respir Dis* **5**, 325-344, doi:10.1177/1753465811406772 (2011).

11 Bowler, R. P. *et al.* Plasma sphingolipids associated with chronic obstructive pulmonary disease phenotypes. *Am J Respir Crit Care Med* **191**, 275-284, doi:10.1164/rccm.201410-1771OC (2015).

12 Nobakht, M. G. B. F., Aliannejad, R., Rezaei-Tavirani, M., Taheri, S. & Oskouie, A. A. The metabolomics of airway diseases, including COPD, asthma and cystic fibrosis. *Biomarkers* **20**, 5-16, doi:10.3109/1354750X.2014.983167 (2015).

**Supplement figure legends**

**
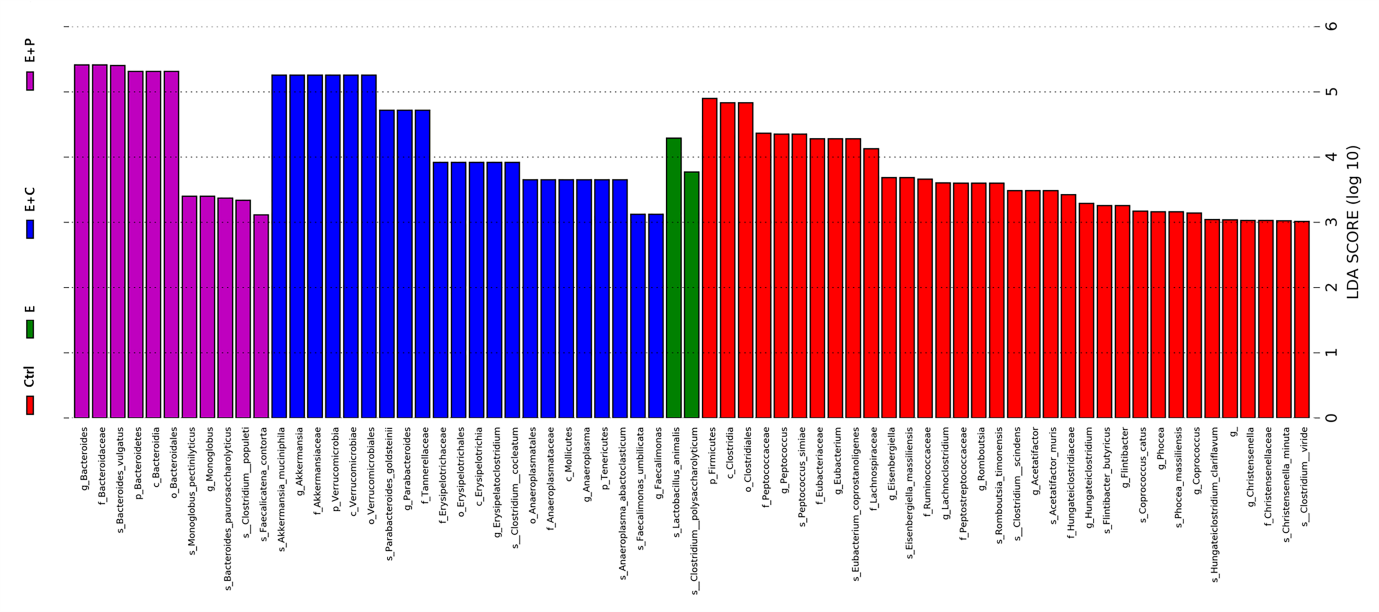
**

**Figure S1.** High-fiber diet alters the composition of the gut microbial community. The histogram of the linear discriminant analysis effect size (LEfSe) analysis for differentially abundant taxa of fecal microbiotas in the four groups; Ctrl (red), control group; E (green), emphysema group; E+C (blue), emphysema with high-cellulose diet group; E+P (violet), emphysema with high-pectin diet group. (n = 5 mice per group).

**
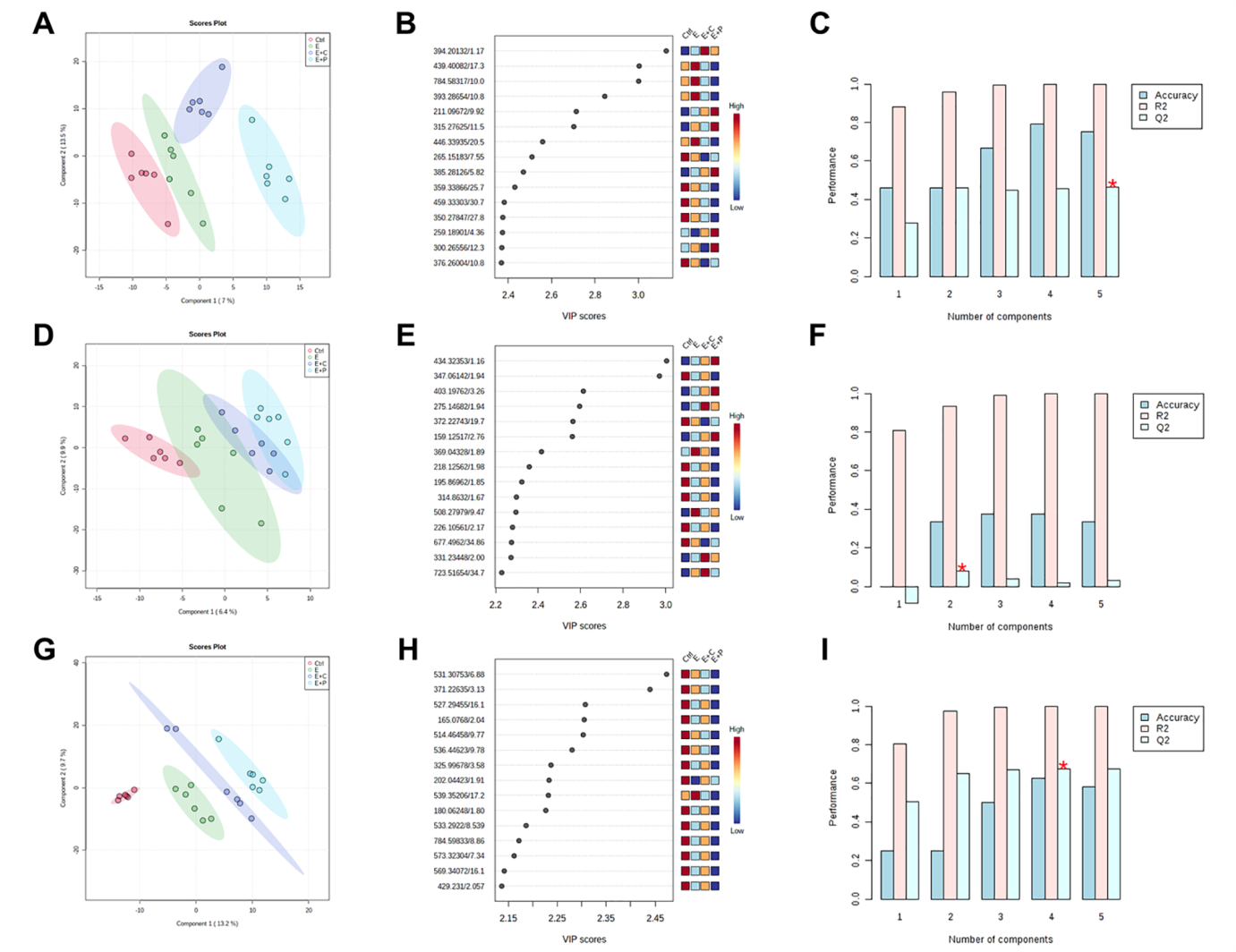
**

**Figure S2.** Summary of pathway analysis with MetaboAnalyst. (**A-I**) Score plots of the PLS-DA. Metabolic features that contributed to the discrimination analysis were shown in variable importance in the projection (VIP) scores. The optimal number of metabolic features for classification was selected for the best Q2, which is the estimate of the predictive ability of the model. (**A-C**) feces, (**D-F**) lung, and (**G-I**) serum. Ctrl, control group; E, emphysema group; E+C, emphysema with high-cellulose diet group; E+P, emphysema with high-pectin diet group.

**
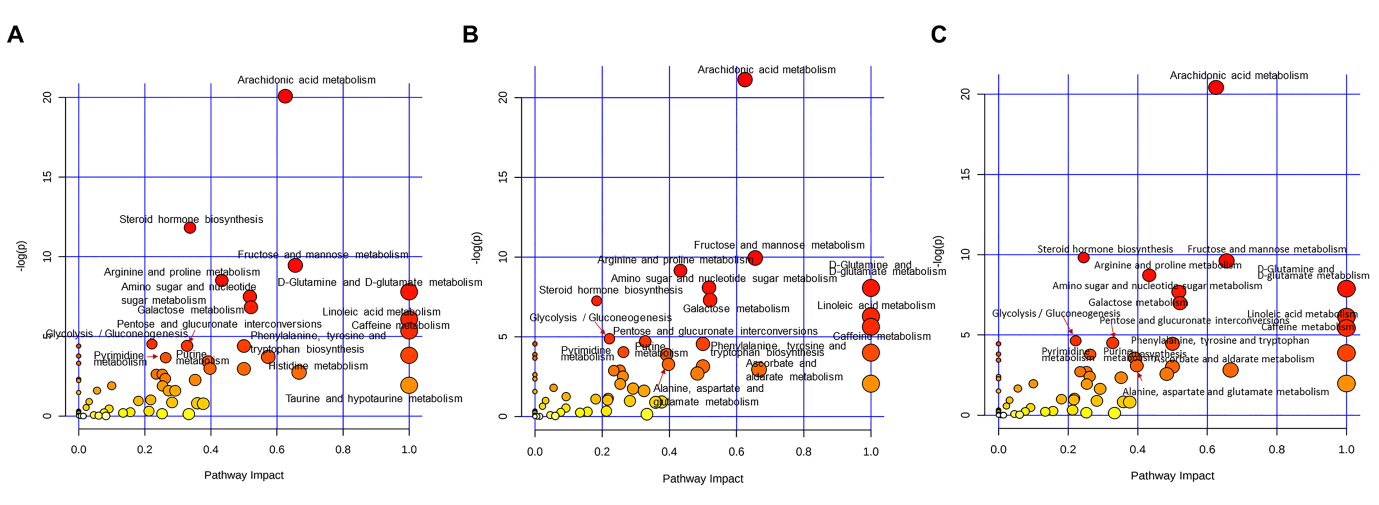
**

**Figure S3.** Summary of pathway analysis with MetaboAnalyst. (**A-C**) Metabolic pathway analysis of the lung in E (**A**), E+C (**B**), and E+P (**C**) were performed with significantly changed metabolic features (*P* < 0.05). Potential target pathways were labeled based on the impact-value threshold (0.10) and *P*-value (0.05). Ctrl, control group; E, emphysema group; E+C, emphysema with high-cellulose diet group; E+P, emphysema with high-pectin diet group.


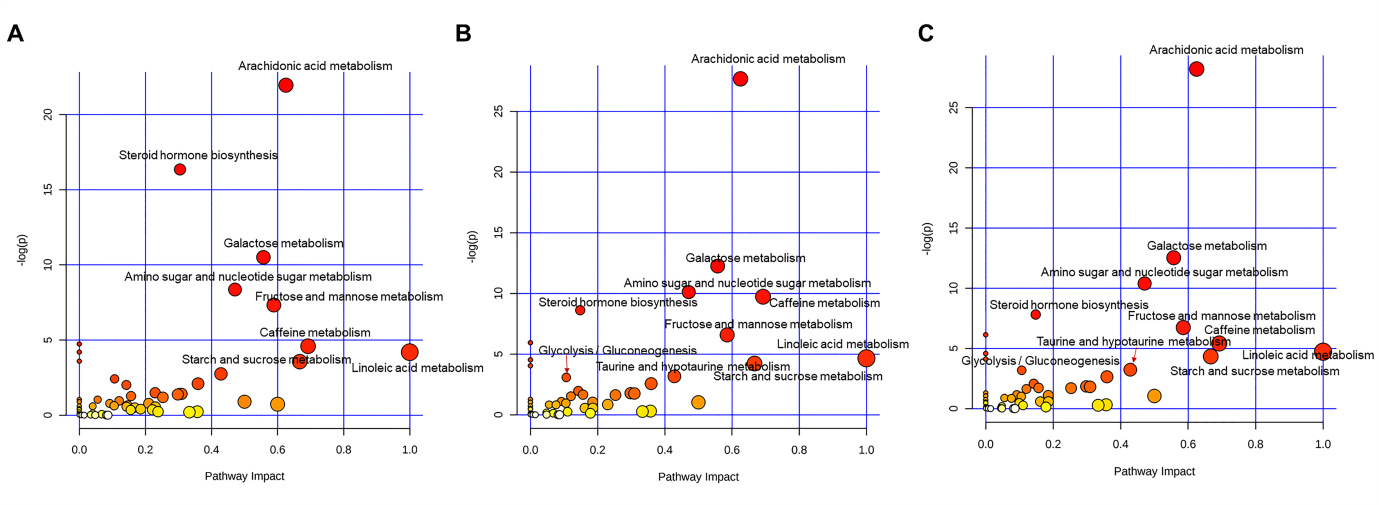


**Figure S4.** Summary of pathway analysis with MetaboAnalyst. (**A-C**) Metabolic Pathway analysis for each of the serum of E (**A**), E+C (**B**), and E+P (**C**) were performed with significantly changed metabolic features (*P* < 0.05). Potential target pathways were labeled based on the impact-value threshold (0.10) and *P*-value (0.05). Ctrl, control group; E, emphysema group; E+C, emphysema with high-cellulose diet group; E+P, emphysema with high-pectin diet group.

**
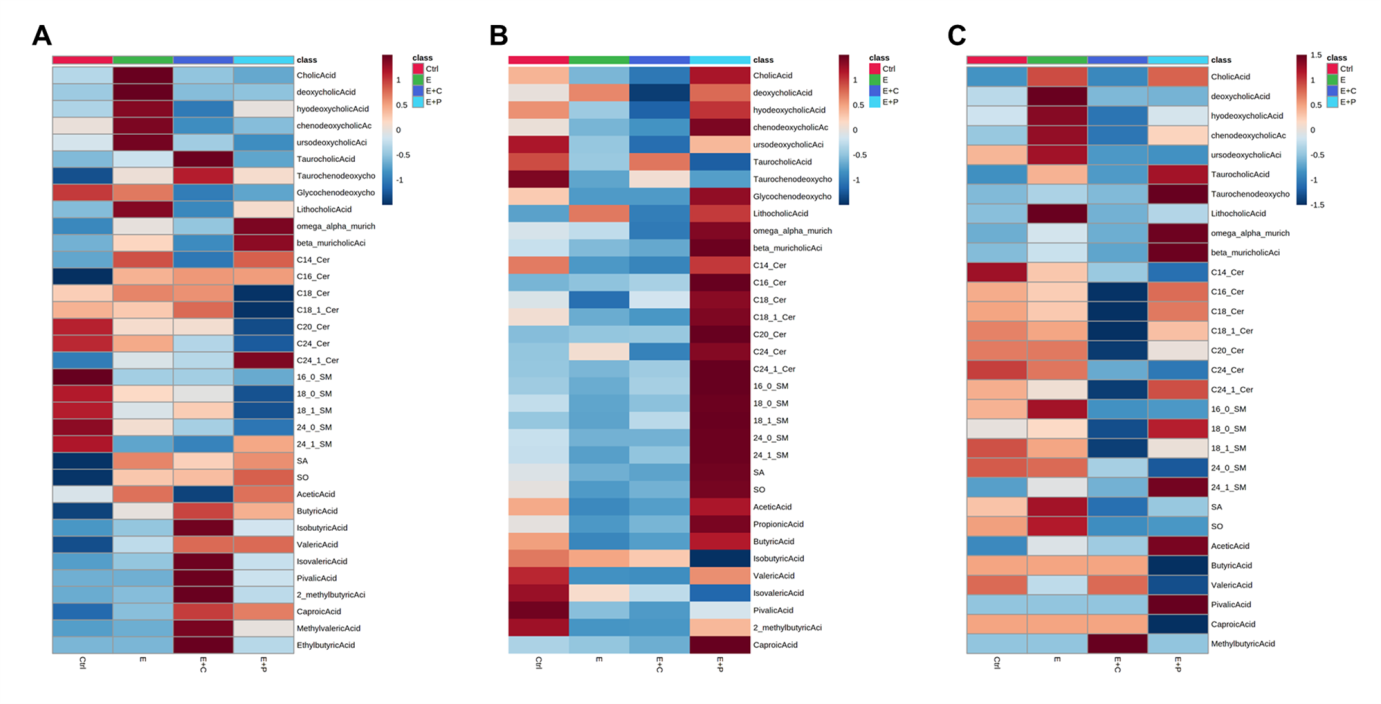
**

**Figure S5.** Metabolic profiles were altered by high-fiber diet intake. (**A-C**) The clustering heatmaps of the top differentially abundant metabolites in the lung (**A**), feces (**B**), and serum (**C**) of the mice in the four groups. (n = 6 mice per group). Ctrl, control group; E, emphysema group; E+C, emphysema with high-cellulose diet group; E+P, emphysema with high-pectin diet group.


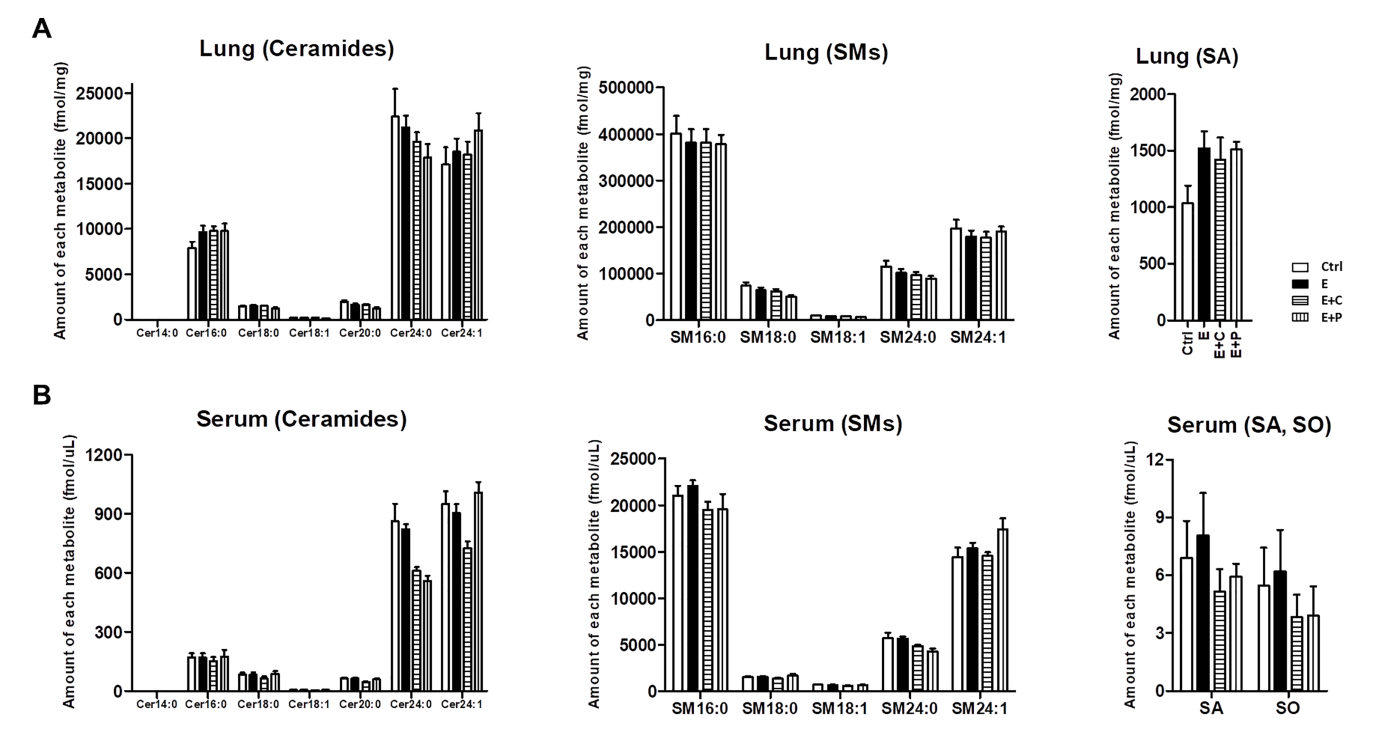


**Figure S6.** Dietary fiber changes the sphingolipid metabolism. The levels of the sphingolipid metabolites in the lung (**A**) and serum (**B**) were measured using metabolite profiling. (n = 6 mice per group). Values are expressed as the mean ± SE. Ctrl, control group; E, emphysema group; E+C, emphysema with high-cellulose diet group; E+P, emphysema with high-pectin diet group; Cer, ceramide, SM, sphingomyelin; SA, sphinganine; SO, sphingosine.

**Table S1**. **Quantitative PCR primer sequences**

| Gene | Forward/reverse | Primer sequence 5′-3′ |
| --- | --- | --- |
| Actin | Forward | \| AAGAGCTATGAGCTGCCTGA \| \| --- \| |
|  | Reverse | \| CACAGGATTCCATACCCAAG \| \| --- \| |
| IFN-γ | Forward | AAGCGTCATTGAATCACACCTG |
|  | Reverse | TGACCTCAAACTTGGCAATACTC |
| IL-1β | Forward | CCAAGCAACGACAAAATACC |
|  | Reverse | GTTGAAGACAAACCGTTTTTCC |
| TGF-β | Forward | CTGCTGACCCCCACTGATAC |
|  | Reverse | GTGAGCGCTGAATCGAAAGC |
| TNF-α | Forward | GACAGTGACCTGGACTGTGG |
|  | Reverse | TGAGACAGAGGCAACCTGAC |
| IL-6 | Forward | TGTGCAATGGCAATTCTGAT |
|  | Reverse | GGTACTCCAGAAGACCAGAGGA |
| IL-8 | Forward | TTGGTGATGCTGGTCATCTT |
|  | Reverse | TTTAGATGCAGCCCAGACAG |
| IL-18 | Forward | GCTGTGACCCTCTCTGTGAA |
|  | Reverse | GGCAAGCAAGAAAGTGTCCT |
| MMP-12 | Forward | GGCCATTCCTTGGGGCTGCA |
|  | Reverse | GGGGGTTTCACTGGGGCTCC |
| IRF-5 | Forward | GCTGGCTACAGGGTTCTGAG |
|  | Reverse | CTGCTGGCTTCATTTCTTCC |
| Cathepsin S | Forward | GCCAGCCATTCCTCCTTCTT |
|  | Reverse | AGCCAACCACAAGAACACCA |
| Bacterial genomic DNA | 341F | CCTACGGGNGGCWGCAG |
|  | 805R | GACTACHVGGGTATCTAATCC |
|  | ITS3 | GCATCGATGAAGAACGCAGC |
|  | ITS4 | TCCTCCGCTTATTGATATGC |

IFN-γ, interferon-γ; IL, interleukin; TGF-β, transforming growth factor-β; TNF-α, tumor necrosis factor-α; MMP, matrix metalloproteinase; IRF-5, interferon regulatory factor-5
